# Plasmid-Mediated Sulfamethoxazole Resistance Encoded by the sul2 Gene in the Multidrug-Resistant *Shigella flexneri* 2a Isolated from Patients with Acute Diarrhea in Dhaka, Bangladesh

**DOI:** 10.1371/journal.pone.0085338

**Published:** 2014-01-09

**Authors:** Mohd S. Iqbal, Mostafizur Rahman, Rafiad Islam, Atanu Banik, M. Badrul Amin, Fatema Akter, Kaisar Ali Talukder

**Affiliations:** 1 Centre for Food and Waterborne Diseases (CFWD), icddr,b, Dhaka, Bangladesh,; 2 Centre for Control of Chronic Diseases (CCCD), icddr,b, Dhaka, Bangladesh; Kliniken der Stadt Köln gGmbH, Germany

## Abstract

In this study, mechanisms of plasmid-mediated sulfamethoxazole resistances in the clinical strains of multi-drug resistant (MDR) *Shigella flexneri* 2a were elucidated for the first time in Bangladesh. From 2006 to 2011, a total of 200 *S. flexneri* 2a strains were randomly selected from the stock of the Enteric and Food Microbiology Laboratory of icddr,b. Antimicrobial susceptibility of the strains showed 73%, 98%, 93%, 58%, 98%, 64% and 4% resistance to trimethoprim-sulfamethoxazole, nalidixic acid, ampicillin, erythromycin, tetracycline, ciprofloxacin and ceftriaxone respectively. Plasmid profiling revealed heterogeneous patterns and interestingly, all the trimethoprim-sulfamethoxazole resistant (SXT^R^) strains yielded a distinct 4.3 MDa plasmid compared to that of the trimethoprim-sulfamethoxazole susceptible (SXT^S^) strains. Curing of this 4.3 MDa plasmid resulted in the susceptibility to sulfamethoxazole alone suggesting the involvement of this plasmid in the resistance of sulfamethoxazole. Moreover, PCR analysis showed the presence of *sul2* gene in SXT^R^ strains which is absent in SXT^S^ strains as well as in the 4.3 MDa plasmid-cured derivatives, confirming the involvement of *sul2* in the resistance of sulfamethoxazole. Furthermore, pulsed-field gel electrophoresis (PFGE) analysis revealed that both the SXT^R^ and SXT^S^ strains were clonal. This study will significantly contributes to the knowledge on acquired drug resistance of the mostly prevalent *S. flexneri* 2a and further warrants continuous monitoring of the prevalence and correlation of this resistance determinants amongst the clinical isolates of *Shigella* and other enteric pathogens around the world to provide effective clinical management of the disease.

## Introduction

Shigellosis is becoming an increasing public health problem due to the emergence of multiple antimicrobial resistances, leading to high rate of global morbidity and mortality especially in the endemic areas like Bangladesh. Approximately 165 million cases of *Shigella* infection occur annually worldwide with 1.1 million deaths with most of the causality are children under 5 years of age [1]. It is well established that *S. flexneri* is the most commonly isolated species in the developing countries; in contrast, *S. sonnei* predominates in developed countries. In developing countries like Bangladesh, the predominant serotype of *S. flexneri* is 2a which is also the most common strain in industrialized countries [1]. Previously efficacious first-line antimicrobial drugs such as sulphonamides, tetracycline, ampicillin, and trimethoprim-sulfamethoxazole have become largely ineffective against prevalent *Shigella* strains in many parts of the world and the recently reported emergence of ciprofloxacin and third-generation cephalosporin resistance, further narrows the choice of effective antimicrobials [2]. The American Academy of Pediatrics and the Infectious Disease Society of America recommend azithromycin as an alternative drug for the treatment of Shigellosis [3]. However, resistance and reduced susceptibility to azithromycin has also been emerged [4], [5]. Thus, the urge for new antibiotics is more pressing than ever. Sulfamethoxazole, a short acting derivative of sulfonamide, is an antibacterial drug widely used since 1930s in the clinical and veterinary medicine to treat bacterial and protozoal infections [6]. The sul2 is one of the three sulfonamide resistance genes and was first identified on a small nonconjugative plasmid of *Escherichia coli* [6]. Plasmid-mediated multidrug resistance is a grave concern for the treatment of infectious diseases. Multiple plasmid-mediated mechanisms of resistance against the fluoroquinolones and aminoglycosides have been described, and the combination of plasmid-mediated resistance with chromosomally encoded resistance mechanisms of multiple drug classes now results in strains that are resistant to all of the main classes of commonly used antimicrobial drugs [7].

In this study, we have characterized 200 clinical MDR *S. flexneri* 2a strains and shown for the first time that the sul2 gene encoded by a small 4.3 MDa plasmid is responsible for the sulfamethoxazole resistance, which eventually will lead to better understanding of effective clinical management of Shigellosis around the world.

## Materials and Methods

### Bacterial strains and serotyping

Two hundred *S. flexneri* 2a strains were randomly selected from patients attending the Dhaka treatment center operated by the International Centre for Diarrhoeal Diseases Research, Bangladesh (icddr,b) in between 2006 and 2011. These strains were isolated and identified in the clinical microbiology laboratory by standard microbiological and biochemical methods [8] and confirmed serologically by using commercially available antisera kit (Denka Saiken, Co. Ltd., Japan) specific for all type- and group-factor antigens as described earlier [8]. A version of *S. flexneri* 2a without the 4.3 MDa plasmid was constructed by curing with acridine orange. *E. coli* strains PDK-9 and V-517 were used as plasmid molecular weight standard [8]. The *Salmonella enterica* serovar Braenderup H9812 was used as molecular size marker for PFGE [8].

### Antimicrobial susceptibility

Bacterial susceptibility to antimicrobial agents was determined as described previously [3] with commercial antimicrobial discs (Oxoid, Basingstoke, UK). The antibiotic discs used in this study were ampicillin (AMP, 10 µg), azithromycin (AZM, 15 µg), ceftriaxone (CRO, 30 µg), chloramphenicol (CHL, 30 µg), ciprofloxacin (CIP, 5 µg), nalidixic acid (NA, 30 µg), sulfamethoxazole (SMX, 25 µg), trimethoprim (TMP, 5 µg), norfloxacin (NOR, 10 µg), trimithoprim/sulfamethoxazole (SXT, 1.25/23.75 µg), streptomycin (STR, 10 µg), tetracycline (TET, 30 µg), mecillinum (MEL, 30 µg), gentamycin (GEN, 10 µg), kanamycin (KAN, 30 µg) and amikacin (AK, 15 µg). *Escherichia coli* ATCC 25922 and *Staphylococcus aureus* ATCC 25923 were used as control strains for susceptibility studies.

### Plasmid profiling

Plasmid DNA was prepared by the simplified alkaline lysis method of Kado and Liu with some modifications as described previously [8]. The molecular mass of the unknown plasmid DNA was assessed by comparison with the mobilities of plasmids with known molecular masses and plasmid DNA of reference *E. coli* strains PDK-9 and V517 were used as molecular mass standards [8].

### Curing of plasmids

The minimum inhibitory concentration (MIC) and sub inhibitory concentration of acridine orange were determined by the agar dilution method [9]. *S. flexneri* 2a strain was grown in Muller-Hinton agar (Becton, Dickinson and Company, USA) plates in the presence of acridine orange (Sigma, St Louis, MO) at variable concentration for 24 h at 37°C. The MIC was defined as the lowest concentration of acridine orange that completely inhibited the visible growth of the test organism after incubation. Plasmid curing was performed as described previously [9]. *S. flexneri* 2a strain was inoculated with Tryptic Soy Broth (TSB) (Becton, Dickinson and Company, USA) containing 0.3% yeast extract in the presence of acridine orange of different concentrations (180, 200, 220, 240 and 260 µg/mL) and then incubated for 24hrs at 37°C. Overnight grown broth culture was then plated on MacConkey agar (Becton, Dickinson and Company, USA) plate and isolated colonies were then subjected to colony patch. Colonies that failed to grow in Tryptic Soy agar (Becton, Dickinson and Company, USA) containing trimethoprim-sulfamethoxazole were considered as putative cured derivatives. Physical loss of the plasmid in the cured derivative was confirmed by agarose gel electrophoresis following extraction of the plasmid DNA from the respective cultures. The percentages of curing efficiency were expressed as the number of colonies with cured phenotype per 100 colonies tested. Antibiotic resistance profiles of plasmid-harboring and plasmid-cured strains were reconfirmed by the disk diffusion method as described earlier [3].

### PCR analysis

Total DNA of SXT^R^, SXT^S^ and 4.3 MDa plasmid cured derivatives of *S.flexneri* 2a strains were prepared according to the procedures described earlier [10]. The *sul1, sul2, sul3, integron1 and integron2* genes were detected by PCR using the primers recommended earlier [11]. Amplifications were performed with a final volume of 30 µl on an thermal cycler (Biorad) with each reaction mixture contained 3.0 µl 5x Green GoTaq Flexi buffer (Promega, USA), 2.0 µl dNTPs (Invitrogen), 10 pmol of each primer, (Integrated DNA Technologies, Inc. USA), 1 µl of total DNA and 1 U of GoTaq Flexi DNA polymerase enzyme (Promega, USA). PCR conditions were adopted from elsewhere [11].

### Pulsed-field gel electrophoresis (PFGE)

PFGE analysis was performed according to the PulseNet standardized protocol [12] using *Xba l* as the restriction enzyme (New England Biolabs, Ipswich, MA). The *Salmonella enterica* serovar Braenderup H9812 was used as molecular size marker [8]. Dendrogram analysis was performed using algorithms available within the Bionumerics software package v.4.5. The unweighted pair group method arithmetic means (UPGMA), with a 2.0% tolerance limit and 1.00% optimization was used to obtain the dendrogram. Strains with a coefficient of similarity ≥90% were considered as genetically closely related.

## Results

### Serotyping and antimicrobial susceptibility testing

All (n = 200) the strains were serologically subtyped and reconfirmed as *S. flexneri* 2a. In order to clarify the association of small plasmids in the drug resistance of *Shigella*, 200 *S. flexneri* 2a clinical strains were subjected to antimicrobial susceptibility testing and found that 73%, 98%, 93%, 58%, 98%, 64% and 4% resistance to SXT, NA, AMP, E, TET, CIP and CRO respectively (Data not shown).

### Plasmid profiling

Plasmid profiling revealed heterogeneous patterns ranging in sizes from approximately 140 to 2.1 MDa among the MDR *S. flexneri* 2a strains. Three plasmids of approximately 140, 2.7 and 2.1 MDa in size were present in more than 90% of the strains and were considered to be the core plasmids of *S. flexneri* 2a (Table 1). Interestingly, all the SXT^R^ (n = 146) strains harbored 4.3 MDa plasmid which is absent in all the SXT^S^ strains (Table 1) and among the eight different plasmid patterns, pattern P1 (140, 4.3, 2.7 and 2.1 MDa) and P6 (140, 2.7, 2.1 MDa) were predominant in SXT^R^ (n = 146) (91.2%) and SXT^S^ (n = 54) (84%) strains respectively (Table 1). The frequency of rest of the patterns was very low.

**Table 1:** Plasmid patterns of MDR *S. flexneri* 2a strains isolated in Dhaka, Bangladesh in between 2006 and 2011.

Serotypes	Plasmid patterns	Plasmid sizes (MDa)	Percentage
SXT resistant strains (n = 146)	P1	140, 4.3,2.7, 2.1	91.2%
	P2	4.3, 2.7, 2.1	4%
	P3	140, 52, 4.3, 2.7, 2.1	3.2%
	P4	105, 4.3, 2.7, 2.1	0.8%
	P5	140, 4.3, 2.7, 2.1, 1.4	0.8%
SXT susceptible strains (n = 54)	P6	140, 2.7, 2.1	84%
	P7	2.7, 2.1	12%
	P8	140, 3.2	4%

### Plasmid curing

**Figure 1:**
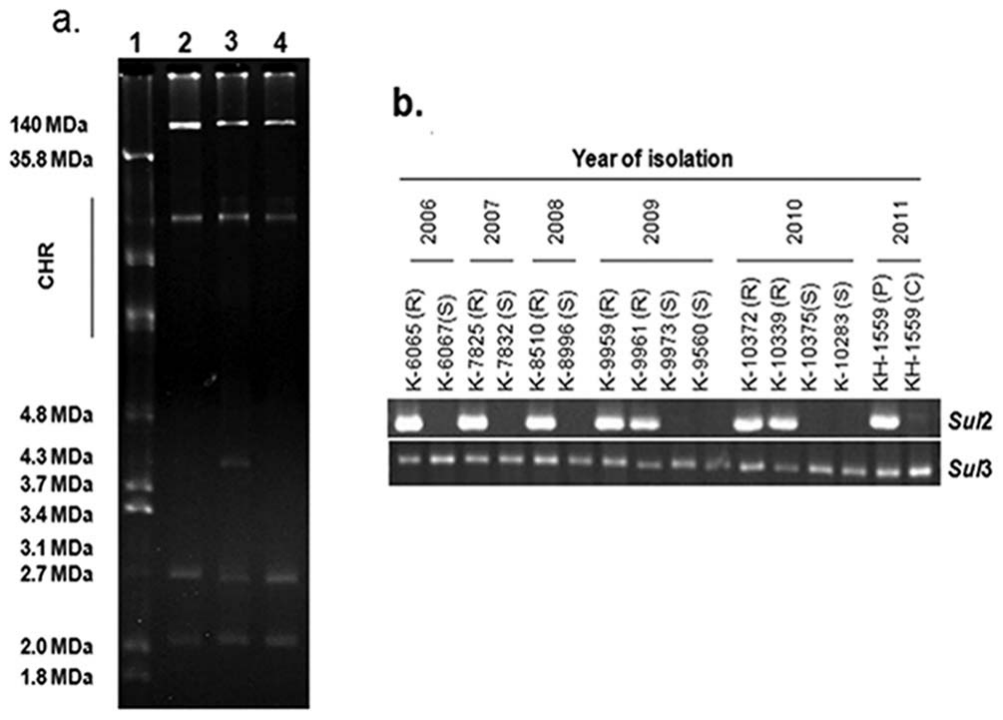
Involvement of 4.3 MDa plasmid and the sul2 gene in the resistance of sulfamethoxazole. **a.** Agarose gel electrophoresis pattern of plasmid DNA isolated from SXT^R^ *S. flexneri* 2a clinical strain KH-1559 and its plasmid cured derivative. Lanes 1 and 2, represents *Escherichia coli* V517 and PDK-9 respectively as size determination markers. Lanes 3 and 4 represent KH-1559(P) SXT^R^ strains and its 4.3 MDa plasmid cured derivative KH-1559 (C) respectively. **b.** *sul2 and sul*3 gene expressions in the representative SXT^R^ (R) and SXT^S^ (S) strains of *S. flexneri* 2a from 2006 to 2011.

Typical in vitro plasmid curing experiments involve exposure of plasmid containing cells to a drug throughout the growth cycle and subsequent assay of the population for the loss of plasmid specific traits. The minimum inhibitory concentration (MIC) of acridine orange against two different clinical strains of MDR *S. flexneri* 2a was found to be 280 µg/ml (Table 2), indicating that acridine orange was not a potent antibacterial agent for the test strains. Acridine orange cured the 4.3 MDa plasmid in the clinical strains of MDR *S. flexneri* 2a (Figure 1a). Curing activity was investigated at different concentrations of acridine orange ranging from 180 µg/mL to 260 µg/mL. The curing efficiency was expressed as the number of colonies with cured phenotype per 100 colonies tested. The highest curing efficiency (3.2%) was observed below the MIC at subinhibitory concentration 260 µg/mL (Table 2).

**Table 2:** Effect of acridine orange concentration on curing efficiency of MDR *S. flexneri* 2a*.

Strain ID	Concentration (µg/ml)	Curing efficiency (%)
**KH 1559**	260	3.20
	240	1.82
	220	0.62
	200	0.13
**KH 1972**	260	2.76
	240	1.34
	220	0.27
	200	0.12

*Five hundred colonies (at each concentration) were tested for curing of the antibiotic resistant phenotype.

### Detection of *sul1, sul2, sul3, integron1* and *integron2* genes in the SXT-resistant and susceptible strains

To investigate the possible mechanisms of SXT resistance, we have checked the presence of *sul1*, *sul2*, *sul3*, *integron1* and *integron2* genes in all the MDR *S. flexneri* 2a strains and found that *sul2* was present in all the SXT^R^ strains (n = 146) and absent in all the SXT^S^ strains (n = 54) (Figure 1b). However, no change was observed in the expressions of *sul1, sul3, integron1 and integron2* genes among the SXT^R^ and SXT^S^ strains. Interestingly, curing of the 4.3 MDa plasmid resulted in the loss of *sul2* and sulfamethoxazole sensitivity in the cured strains confirming the involvement of 4.3 MDa plasmid and *sul2* in the resistance of sulfamethoxazole (Figure 1 and Table 3), as has also been supported by others. [6], [13]–[15].

**Table 3:** Antibiogram of two MDR *S. flexneri* 2a strains and their 4.3 MDa plasmid-cured derivatives*.

Antimicrobial agents	Diameter of inhibition zone (mm)			
	Before curing		After curing	
	Resistance	Susceptibility	Resistance	Susceptibility
Ampicillin (AMP)	8	-	8	-
Azithromycin (AZM)	-	25	-	26
Ceftriaxone (CRO)	-	36	-	35
Chloramphenicol (C)	12	-	11	-
Ciprofloxacin (CIP)	-	30	-	30
SXT	8	-	-	27
Sulfamethoxazole(SMX)	8	-	-	25
Trimethoprim (TMP)	8	-	8	-
Amikacin (AK)	-	22	-	21
Nalidixicacid (NA)	8	-	8	-
Gentamicin (GEN)	-	25	-	24
Kanamycin (KAN)	-	25	-	26
Norfloxacin (NOR)	-	30	-	29
Streptomycin (STR)	8	-	8	-
Tetracycline(TET)	10	-	10	-
Mecillinam (MEL)	-	26	-	27

SXT, trimethoprim-sulfamethoxazole (Co-trimoxazole).

*Wild-type MDR *S. flexneri* 2a strains were resistant to eight antibiotics and susceptible to eight; the plasmid-cured derivatives showed that the antibiotic resistance loci of sulfamethoxazole were plasmid-encoded.

### Pulsed-field gel electrophoresis (PFGE)

**Figure 2:**
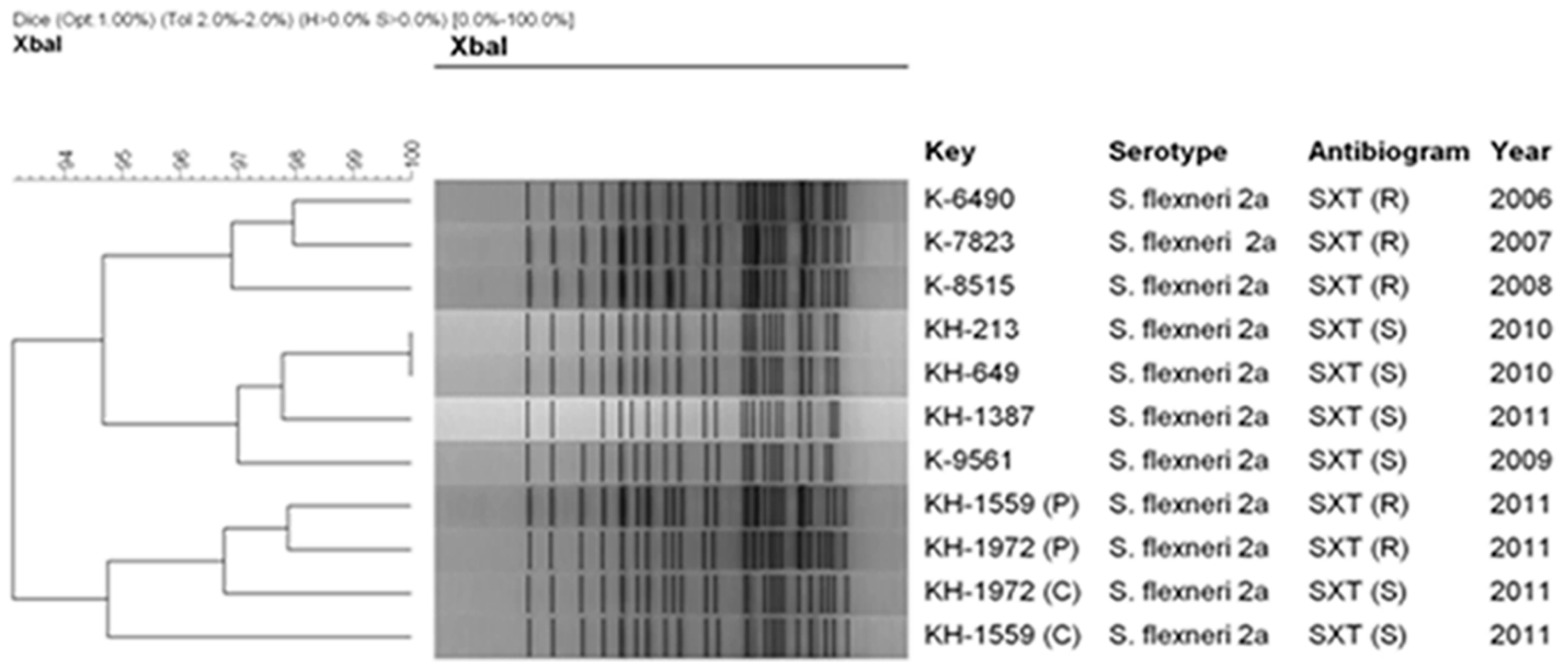
PFGE patterns of representative SXT^R^ (R) and SXT^S^ (S) *S. flexneri* 2a strains and their plasmid cured (C) derivatives. The dendrogram was constructed with Bionumerics v.4.5 software using the unweighted pair group method with arithmetic means (UPGMA). Dendrogram analysis showed that the plasmid cured strains and the parent strains are very close (95%) in their homology.

PFGE has been used for the identification of new strains within a community and determining the clonal diversity among the circulating pathogens [8]. PFGE analysis of the *Xba*I-digested chromosomal DNA of the MDR *S. flexneri* 2a strains and their plasmid cured derivatives yielded DNA fragments ranging in size approximately from 20 to 670 Kb. Dendrogram analysis exhibited the same PFGE patterns among the representative SXT^R^ and SXT^S^ strains indicating that they have originated from a common ancestor (Figure 2). Besides, both the parent and 4.3 MDa plasmid cure derivatives were very much close in their homology (95%) demonstrating no effect of acridine orange treatment on chromosomal DNA (Figure 2).

## Discussion

The emergence and dissemination of multi-drug resistant strains of *Shigella* are particularly of greater importance in developing countries like Bangladesh due to the accessibility of multiple drugs which further augments the resistance rate resulting in frequent treatment failures. Our study demonstrates an increasing incidence of antimicrobial resistance of the mostly prevalent *S. flexneri* 2a serotype isolated in between 2006 to 2011 and also determines a 4.3 MDa plasmid-mediated sulfamethoxazole resistance encoded by the sul2 *gene*. Moreover, these MDR strains were concurrently resistant to ≥3 of the eight antimicrobial agents used and were detected in 95% of the *S. flexneri* 2a isolates. Resistance to ampicillin + nalidixic acid + trimethoprim-sulfamethoxazole was the most common pattern whereas resistances to at least four, five and six antimicrobial agents were found to be 92%, 77% and 33% respectively. None of the strains were found to be resistant to at least seven or eight antibiotics. Alarmingly, significant increase (32%) in the multi-drug resistance was observed among the *S. flexneri 2a* strains as compared to that of the 2001–2002 isolates reported by Rahman M et al. [5]. This increased resistance phenomena could be explicated by widespread and indiscriminate use of antibiotics over longer periods to treat Shigellosis, resulting in the positive selection pressure and maintenance of drug resistance [16]. Almost all the strains from 2010 were found to be resistant (95%) to ciprofloxacin as has also been found in neighboring Kolkata, India during 2007–2009, where the resistance was 91.6%. Moreover, similar trend has been reported in many South Asian countries [3]. Interestingly, almost all the tested strains from 2009 and 2010 were susceptible to mecillinam and thus currently used as an empirical drug for the treatment of Shigellosis in Bangladesh [5]. Plasmid profiling could be an attractive tool in epidemiological investigations of various enteric pathogens [17]. Our study revealed heterogeneous populations of plasmids in *S. flexneri* 2a strains ranging from two to five in numbers. Of these 200 strains, 12% lost the large, 140 MDa plasmid, mostly known for the invasiveness of the bacteria and possibly due to prolonged storage at −70°C and repeated sub-culturing as has also been described by Vargas et al. [18]. In addition to the large plasmid, about 98% of the strains contained two plasmids of approximately 2.7 MDa and 2.1 MDa in size and these two along with the 140 MDa plasmid are considered as the core plasmid (140, 2.7 and 2.1 MDa) of *S. flexneri* 2a as reported earlier [19]. Interestingly, all the SXT^R^ (n = 146) strains harbored a unique 4.3 MDa plasmid which is absent in all the SXT^S^ (n = 54) strains and paved us the way towards further investigation (Table 1). Curing of this 4.3 MDa plasmid from two of the recently isolated SXT^R^ strains showed resistance to six drugs (AMP, C, NA, TMP, STR, TET) and susceptibility to ten drugs (SXT, SMX, AZM, CRO, CIP, NOR, MEL, GEN, KAN, AK). In contrast, wild type strains were resistant to SXT, SMX, TMP AMP, C, NA, STR and TET (Table 3) and thus confirming the involvement of 4.3 MDa plasmid in the resistance to sulfamethoxazole alone as has also been reported elsewhere [9]. In gram-negative clinical isolates, spread of antibiotic resistance determinants by integrons underlies the rapid evolution of MDR phenotypes [20]. Both *sul1* and *sul3* genes are thought to be associated with class 1 integrons while the sul2 with the class 2 integrons [11], [20]. In our study, sulfamethoxazole resistance observed in *S. flexneri* 2a strains was conferred by the sul2 gene encoded by a small non-conjugative plasmid as reported earlier [9], [21]. However, apart from *sul2*, no significant difference was observed in *integron1*, *integron2*, *sul1* and *sul3* gene expressions among all the 200 strains irrespective of SXT resistance which might be attributable to possible promoter inactivation or other environmental factors. Similarly, Hu LF and colleagues showed the involvement of 7.3 kb plasmid encoded the sul2 gene conferring high SXT resistance in the clinical isolates of *Stenotrophomonas maltophilia* [13].

In conclusion, this study collectively demonstrates for the first time the association of *sul2* and 4.3 MDa plasmid in the sulfamethoxazole resistance of the MDR clinical strains of *S. flexneri* 2a in Bangladesh. Further analysis of the function of small plasmids as well as the comparative analysis of virulence plasmids from other *Shigella* serotypes will allow a more complete characterization of the evolution of these plasmids. Although, more severe approaches should be taken to clarify the precise molecular mechanism; this study will significantly contributes to the knowledge on acquired drug resistance of the mostly prevalent *S. flexneri* 2a and thereby warrants continuous monitoring of the prevalence and correlation of this resistance determinants amongst the clinical isolates of *Shigella* and other enteric pathogens around the world to provide effective clinical management of the disease.
